# The structure of innate vocalizations in *Foxp2*-deficient mouse pups

**DOI:** 10.1111/j.1601-183X.2010.00570.x

**Published:** 2010-06

**Authors:** S Gaub, M Groszer, S E Fisher, G Ehret

**Affiliations:** † Institute of Neurobiology, University of UlmUlm, Germany; ‡ Wellcome Trust Centre for Human Genetics, University of OxfordRoosevelt Drive, Oxford, UK; § Institut du Fer à Moulin, INSERM U839, rue du Fer à MoulinParis, France

**Keywords:** Acoustical communication, *Foxp2* mutation, instinctive vocalizing, motor control, mouse calls, speech disorder, vocalization deficits

## Abstract

Heterozygous mutations of the human *FOXP2* gene are implicated in a severe speech and language disorder. Aetiological mutations of murine *Foxp2* yield abnormal synaptic plasticity and impaired motor-skill learning in mutant mice, while knockdown of the avian orthologue in songbirds interferes with auditory-guided vocal learning. Here, we investigate influences of two distinct *Foxp2* point mutations on vocalizations of 4-day-old mouse pups (*Mus musculus*). The *R552H* missense mutation is identical to that causing speech and language deficits in a large well-studied human family, while the *S321X* nonsense mutation represents a null allele that does not produce Foxp2 protein. We ask whether vocalizations, based solely on innate mechanisms of production, are affected by these alternative *Foxp2* mutations. Sound recordings were taken in two different situations: isolation and distress, eliciting a range of call types, including broadband vocalizations of varying noise content, ultrasonic whistles and clicks. Sound production rates and several acoustic parameters showed that, despite absence of functional Foxp2, homozygous mutants could vocalize all types of sounds in a normal temporal pattern, but only at comparably low intensities. We suggest that altered vocal output of these homozygotes may be secondary to developmental delays and somatic weakness. Heterozygous mutants did not differ from wild-types in any of the measures that we studied (*R552H*) or in only a few (*S321X*), which were in the range of differences routinely observed for different mouse strains. Thus, *Foxp2* is not essential for the innate production of emotional vocalizations with largely normal acoustic properties by mouse pups.

Heterozygous mutations of human *FOXP2* cause severe speech and language disorders (Lai *et al*. 2001; MacDermot *et al*. 2005) involving deficits in rapid coordinated orofacial movements and impaired expression and reception of language (Watkins *et al*. 2002). Neuroimaging and behavioural data suggest changes in frontostriatal and/or frontocerebellar brain circuits mediating learning, planning and execution of orofacial motor sequences (Vargha-Khadem *et al*. 2005). *FOXP2* encodes a transcription factor (Vernes *et al*. 2007) found in highly similar form and expressed in corresponding brain areas in many vertebrates, including rodents and birds (Fisher & Scharff 2009). Its involvement in human spoken language may be built on evolutionarily ancient circuits implicated in sensory–motor functions and motor-skill learning (Fisher & Marcus 2006). Lentiviral-based knockdown of *FoxP2* expression in a key striatal nucleus of juvenile zebrafinches during song learning yields inaccurate and incomplete vocal imitation (Haesler *et al*. 2007). Heterozygous mice with disrupted *Foxp2* show abnormal synaptic plasticity in relevant circuits and impaired motor-skill learning (Groszer *et al*. 2008). Whether and how their vocalizations are changed remains to be clarified in view of differences in data (Fujita *et al*. 2008; Groszer *et al*. 2008; Shu *et al*. 2005).

To understand *FOXP2/Foxp2* functions, it is necessary to distinguish roles in innate versus learned complex movement patterns. In the present study, we address this by analysing vocalizations of mouse pups carrying aetiological *Foxp2* mutations. *Foxp2-R552H* mutants carry an arginine-to-histidine substitution in the DNA-binding domain of the encoded protein, matching a human *FOXP2-R553H* mutation which impairs speech and language in a large family (Lai *et al*. 2001) and disrupts protein function (Vernes *et al*. 2006). Here, we significantly extend previous vocalization analyses of these mutants (Groszer *et al*. 2008), incorporating larger numbers of animals and novel sound parameters, and perform parallel investigations of a second distinct line with aetiological relevance. *Foxp2-S321X* mice carry an early stop codon at position 321 of the protein, close to a human *FOXP2-R328X* mutation disturbing speech and language in another family (MacDermot *et al*. 2005). The human/mouse nonsense mutations are likely to represent null alleles (Groszer *et al*. 2008; MacDermot *et al*. 2005; Vernes *et al*. 2006).

Unlike prior investigations of *Foxp2* disruption on mouse pup ultrasound emission at 6–10 days of age (Fujita *et al*. 2008; Shu *et al*. 2005), we characterize three vocalization types – ultrasonic whistles, clicks and harmonically structured calls of various frequency bandwidths containing varied amounts of noise – which are innately produced by 4-day-old animals in states of isolation (ultrasounds and clicks) and distress (harmonic sounds, ultrasounds and clicks) (Ehret 1975; Haack *et al*. 1983). Pups are deaf up to the age of 9 days (Ehret 1976), lacking auditory feedback which may influence the intensity, pitch and variability of calls (Romand & Ehret 1984). Thus, we establish the influence of *Foxp2* mutations on emotionality and motor coordination of early vocalizations without intervening auditory feedback and learning. Compared with data on humans and songbirds, we separate possible impacts of *Foxp2* mutations on innate motor coordination from effects on motor-skill learning.

## Materials and methods

### Animals

*Foxp2-R552H* and *Foxp2-S321X* mutant mice were generated via a gene-driven *N*-ethyl-*N*-nitrosourea (ENU) mutagenesis strategy. As previously described by Groszer *et al*. (2008), the founders were crossed onto the C3H/HenNHsd background for up to nine generations, exploiting marker-assisted backcrossing to accelerate homogenization of genomic background and elimination of non-relevant ENU mutations. We then paired heterozygous females of both *Foxp2* mouse lines (*R552H* and *S321X*) with heterozygous males of the same lines. The pairs were housed in plastic cages (26.5 × 20 × 14 cm) at an average temperature of 23°C and a 12-h light–dark cycle (light on at 8 h). Wood shavings served as nest material. Food and water were available *ad libitum*. To allow both mating in the postpartum oestrus of the female and an undisturbed raising of the pups by the female, the male was removed at the day of birth of the pups. *R552H* heterozygous females had 3–10 pups and *S321X* heterozygous females 3–9 pups in their litters. The tested animals, 12 pups of each genotype (wild-type, heterozygous and homozygous for the *Foxp2-R552H* or *Foxp2-S321X* mutation), were selected from 10 *R552H* and 9 *S321X* litters. Thus, we set out with recording and analysing vocalizations from 6 ×12 = 72 mouse pups. In the figures, groups of wild-type animals are indicated by +/+, of heterozygotes by *R552H*/+ or *S321X*/+ and homozygotes as *R552H*/*R552H* or *S321X*/*S321X*. Experiments were carried out in accordance with the European Communities Council Directive of 24 November 1986 (86/609/EEC) and were approved by the Regierungspräsidium Tübingen (Germany).

### Genotyping

Mice were genotyped by polymerase chain reaction (PCR) and restriction digestion of genomic DNA. For the *R552H* line, the following primers were used: 5′-GTTCCTCTGGACATTTCAAC-3′ and 5′-TGTGAGCATGCCTTTAGCTG-3′. PCR conditions were as follows: 94°C for 1 min (1 cycle), 94°C for 30 seconds, 55°C for 30 seconds, 68°C for 1 min (35 cycles) and 72°C for 10 min (1 cycle). The 603 bp PCR products were digested with *Hga*I which yields fragments of 372 and 231 bp for the wild-type allele, while the mutant *R552H* allele remains undigested.

For *S321X*, the following primers were used: 5′-ATAGTATGGAAG ACAACGGCATC-3′ and 5′-GATGGGGTTAGTGAATGTTCTCA-3′. PCR conditions were as follows: 95°C for 15 min (1 cycle), 94°C for 1 min, 55°C for 1 min, 72°C for 1 min (35 cycles) and 72°C for 10 min (1cycle). The 468 bp PCR products were digested with *Afl*II, which yields fragments of 332 and 136 bp for the mutant *S321X* allele, while the wild-type allele remains undigested.

### Recording of pup vocalizations

Vocalizations of the mouse pups were recorded at day 4 after birth. We selected this developmental time-point for three important reasons. First, at this age, the production rate of ultrasounds during activity periods under isolation conditions becomes maximal (Haack *et al*. 1983; Okon 1970). Second, because the pups are still deaf, their vocalizations reflect innately specified production mechanisms, without any influence of auditory feedback. Third, the selection of an early time-point minimizes potential effects of postnatal developmental delays in mutants, which could act as confounding factors (Groszer *et al*. 2008).

The recordings were performed in a soundproof and anechoic room under dim red light at an average temperature of 23°C. For recording of ultrasounds in response to isolation (USIs), a pup was separated from its mother and littermates and placed in a round and shallow dish (diameter: 14 cm, height: 4.5 cm). Usually, pups started emitting USIs shortly after being isolated. Most USIs were recorded during motor activities (righting, falling over and pivoting) of aroused pups as described by Haack *et al*. (1983). After 15 min of recording possible USIs, the pup was lifted and its tail gently pressed between thumb and index finger for releasing distress calls (DCs), which were audible to the human observer, for a minimum of 10 seconds. In this situation of DC production, pups also vocalized ultrasounds (Ehret 1975) that were called ultrasounds under distress (USDs). Vocalizations were recorded with a calibrated condenser microphone (Bruel and Kjaer, Model 4135) with preamplifier (Bruel and Kjaer, Model 2633) positioned about 8 cm away from the pup's mouth. The output of the microphone was high-pass filtered (Kemo VBF 10M, 132 dB/octave, 20 kHz high-pass for USIs, 500 Hz high-pass for DCs and USDs), amplified (Bruel and Kjaer measuring amplifier, Model 2636, 40 dB setting for USIs and 70 dB setting for DCs and USDs) and recorded (Toshiba notebook CPU, 500 kHz DAQCard-6062E National Instruments, signal software version 4.1; Engineering Design, Berkeley, CA, USA) with a gain of 10.0 and a sampling rate of 357143 Hz. Under these recording conditions, overload of the equipment or clipping of the recordings did not occur. After recording of the vocalizations, the pup was weighed, individually marked for identification and genotyped as described above.

Analyses of the acoustical parameters of the vocalizations were performed with signal software versions 4.0 and 4.1. In order to measure sound pressure levels (SPLs) of the recorded vocalizations, a calibration procedure was necessary. For this, a synthesized tone of a frequency of 20 kHz was recorded as described above for the DCs, USDs and USIs. At the same time, the peak SPL of the tone was read from the display of the measuring amplifier and noted. The SPLs were adjusted to 60 dB for USIs and 85 dB for DCs and USDs, respectively, and the rms-voltages of the recordings corresponding to these SPL values were noted. With this calibration, the voltages of the recorded pup calls were calculated in dB SPL.

The recordings and analyses of pup calls were carried out by the experimenters blind for the genotype of the pups.

### Analysis of sound parameters

The following parameters of the pup calls were analysed: number of emitted calls, number of clicks associated with production of USIs and USDs, number of USIs and USDs with frequency jumps, noise content of DCs, peak SPL, call duration and duration of inter-call intervals in series of USIs and DCs.

### Statistical analysis

The statistical analyses were carried out with SigmaStat software (version 3.1). Normally distributed data were plotted in the figures as means ± standard deviation. The means of two groups were compared with the *t* test, of several groups with a one-way analysis of variance (anova) followed by the Tukey test for comparisons between two groups. Where data were not normally distributed (assessed by Kolmogorov–Smirnov test), they were plotted in the figures as medians plus 25%, 75% quartiles and range. Two groups of such data were compared with the *U* test and several groups with a one-way anova on ranks followed by the Dunn's test for comparisons between two groups. If the Dunn's test showed significant differences (*P* < 0.05, as indicated in the SigmaStat program), then *U* tests were conducted to assess the final significance values between two groups. These are indicated in the figures. Further, numbers of alternative events (e.g. numbers of calling or non-calling animals of different genotypes) were compared with the chi-square test. All statistical tests were two-tailed, with α≤ 0.05. Significant differences, as emerging from the above-mentioned tests, are indicated in the figures by *P≤ 0.05, **P≤ 0.01 and ***P≤ 0.001. Table S1 in the supplement provides an overview of the statistical tests applied to the data shown in the figures together with the parameters and the obtained results.

## Results

### General

**Figure 1:**
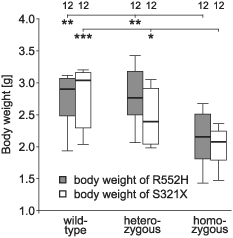
Body weight of pups of both *FOXP2* mouse lines (*R552H* and *S321X*) Homozygous mutants of both lines weighed significantly less compared with their wild-type and heterozygous littermates. The number of animals per group is shown in the top row of the figure.

Consistent with Groszer *et al*. (2008), homozygous pups of *R552H* and *S321X* mouse lines displayed developmental delays and severe motor impairments, surviving only 3–4 weeks after birth. There was evidence of reduced body weight compared with wild-types and heterozygotes already at postnatal day 4 (anova on ranks, *P* < 0.01 for the *R552H* line and *P* < 0.001 or *P* < 0.05 for the *S321X* line; Fig. 1). All heterozygotes were fully viable and overtly normal (also see Groszer *et al*. 2008).

In the isolation situation, USIs were emitted by all wild-type and heterozygous animals and by 4 of the 12 *S321X* homozygotes. No USIs could be recorded from the *R552H* homozygotes. Thus, there were significantly lower numbers of USIs from homozygotes of both mouse lines compared with the respective heterozygotes and wild-types (chi-square test, *P* < 0.001 in each case), while significantly more *S321X* homozygotes produced USIs compared with *R552H* homozygotes (chi-square test, *P* = 0.03).

**Figure 2:**
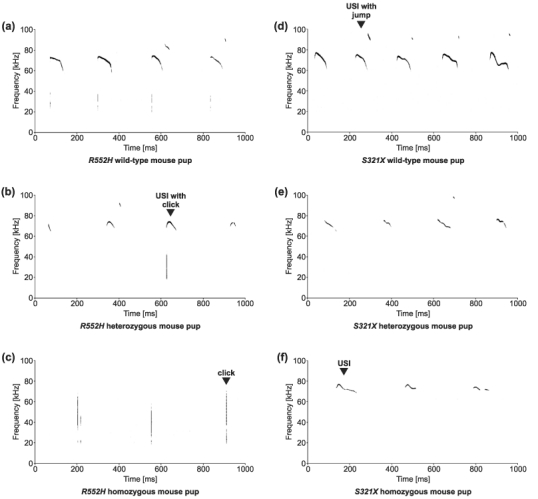
Example spectrograms of series of ultrasounds (USIs) and clicks from isolated pups The genotype of the vocalizing pup is indicated below the spectrograms. (a, b) USIs, partly with clicks; (c) clicks without USIs. Some USIs (a, b, d, e) end with a frequency jump.

Example spectrograms of USI series are shown in Fig. 2. All USIs were frequency modulated pure-tone whistles, mostly in the frequency range between 60 and 80 kHz. Some of the USIs showed a frequency jump (Fig. 2a,d) mainly at the end of the USI. USIs often started with a click, which is a short broadband noise pulse (examples in Fig. 2a,b). Although no USIs were detected from *R552H* homozygotes, all animals produced series of clicks (Fig. 2c).

**Figure 3:**
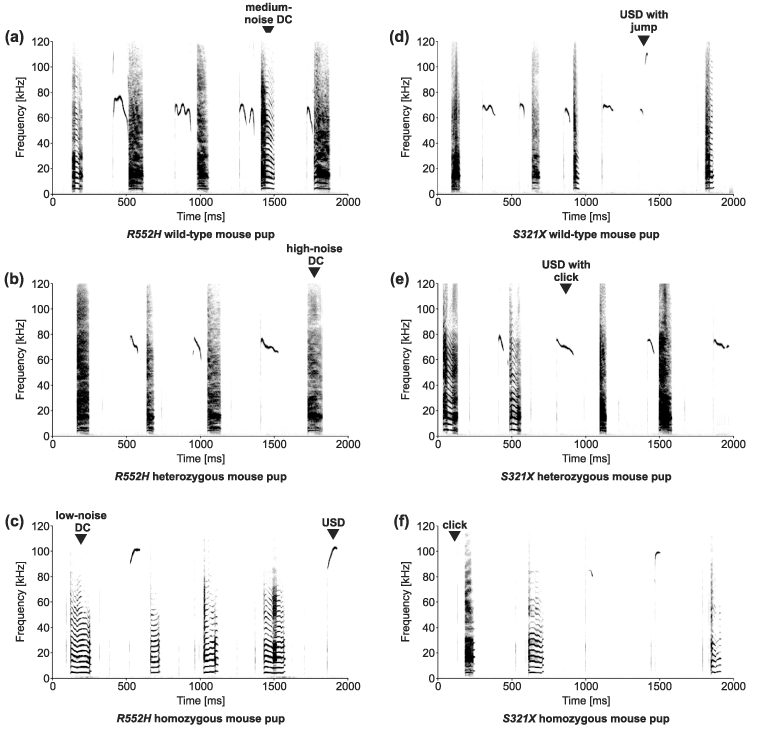
Example spectrograms of DCs alternating with USD and clicks The genotype of the vocalizing pup is indicated below the spectrograms. The DCs may show a high-noise content (high-noise DC), a medium-noise content (medium-noise DC) or a low-noise content (low-noise DC). USDs may start with a click and may end with a frequency jump.

In the distress situation, all animals in all groups vocalized series of DCs interspersed with USDs and clicks (Fig. 3). Like USIs, USDs were frequency modulated pure-tone whistles, and many started with a click. In a similar manner to USIs, some USDs showed a frequency jump, mainly at the end of the call. The DCs were broadband vocalizations covering a frequency range between about 3 and 120 kHz. To gain more insight into call structures, we measured the noise content in spectrograms as spectral energy occurring over periods of the vocalizations in between or instead of harmonic components (Fig. 3). An evaluation of all analysed DCs showed that the distribution of the number of DCs with different percentages of noise content split into three classes: the number of DCs with no noise content (0–5%) was lowest. Then, the number of DCs with a higher noise content increased up to a noise content of 20–25% followed by a sharp decrease in the number of DCs with a noise content of 25–30%. Then, the number of DCs with a higher noise content again increased up to a noise content of 55–60% followed by a sharp decrease in the number of DCs with a noise content of 60–65%. Finally, there was a high number of DCs with a noise content up to 80–100%. Therefore, we defined low-noise DCs as those containing noise for up to 25% of their duration, medium-noise DCs as those containing noise for 25–60% of their duration and high-noise DCs as those containing noise for more than 60% of their duration. Example vocalizations are indicated in Fig. 3. In many of the high-noise DCs, the harmonic frequency structure of the calls was replaced by the noise (Fig. 3).

### Numbers of vocalizations

**Figure 4:**
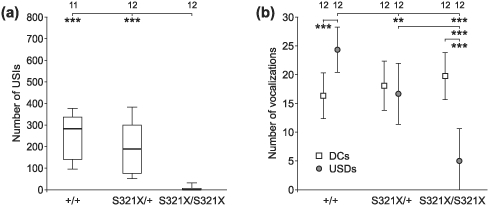
Number of vocalizations emitted by S321X mutants (a) USIs. Wild-types and heterozygotes produced similar USI rates during the 15 min of recording, while only 4 of the 12 tested homozygotes vocalized under these conditions, producing just a few USIs. (b) Number of DCs and USDs emitted in the distress situation. The rates of DCs did not depend on the genetic background. The ratio of USDs to DCs varied among the different groups, with lowest numbers of USDs emitted by homozygotes. Data for *R552H* lines are shown in Fig. S1. Sounds of 11 or 12 animals per group could be analysed (see numbers in the top row of the figure).

**Figure 5:**
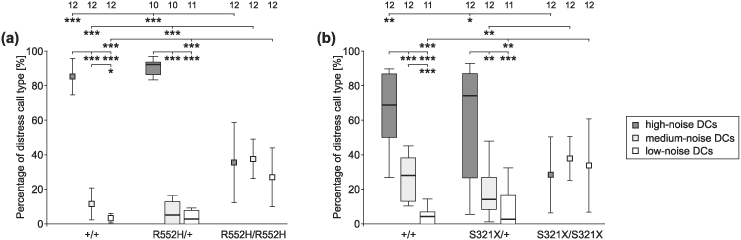
Percentages of the emitted DC types according to their noise content In homozygotes of both mouse lines [(a)*R552 H* and (b)*S321 X*], DCs of high-, medium- or low-noise content occurred at similar rates of about 30%. Wild-type and heterozygous animals of both mouse lines produced more high-noise than medium-noise or low-noise DCs; the wild-types also more medium-noise than low-noise DCs. Sounds of 10–12 animals per group could be analysed (see numbers in the top row of the figure).

In line with our previous analyses of *R552H* mutants (Groszer *et al*. 2008; anova on ranks, *P* < 0.001; Fig. S1), wild-types and heterozygotes of the *S321X* line vocalized significantly more USIs (anova on ranks, *P* < 0.001; Fig. 4a) and USDs (anova, *P* < 0.001; Fig. 4b) than homozygotes. *S321X* wild-types produced significantly more USDs than heterozygotes (anova, *P* < 0.01; Fig. 4b). The number of DCs did not differ among the three genotypes of either mouse line (Fig. 4b, Fig. S1b), i.e. homozygous *S321X* and *R552H* animals vocalized, on average, as many DCs as their respective heterozygous and wild-type littermates. Significant differences between the genotypes occurred, however, with regard to the distribution of the DCs with different noise content. Wild-types and heterozygotes of both mouse lines produced significantly more high-noise DCs than medium- and low-noise DCs (anova or anova on ranks, *P* < 0.001 or *P* < 0.01) while in the homozygotes, DCs of the three classes occurred about equally frequently (Fig. 5). Thus, homozygous mutants consistently produced less high-noise and more low-noise DCs than the wild-types and heterozygotes (for the *R552H* line anova on ranks, *P* < 0.001; for the *S321X* line anova on ranks, with *P* < 0.001, *P* < 0.01 or *P* < 0.05; Fig. 5).

**Figure 6:**
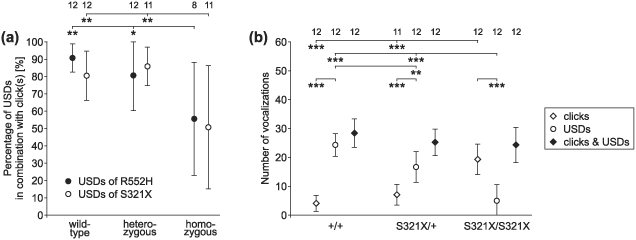
Ultrasounds (USDs) and clicks produced by *R552H* and *S321X* mutants in the distress condition (a) About 80% of the USDs from the wild-type and heterozygous animals of both mouse lines contained clicks, while only about 50% of the USDs from the respective homozygotes had clicks. Percentage of USIs with clicks is shown in Fig. S3. (b) Wild-type and heterozygous pups emitted more USDs than clicks, while this was reversed in homozygotes. The sum of the emitted clicks and USDs, however, did not differ between genotypes. Data for *R552H* lines are shown in Fig. S3. Sounds of 8 to 12 animals per group could be analysed (see numbers in the top row of the figure).

Because ultrasounds may start with a click (Figs 2 and 3), we compared the percentage of USIs and USDs with clicks among the genotypes. Wild-type and heterozygous animals of both mouse lines vocalized similar percentages (20–40% on average, no statistically significant differences, *t* test) of USIs with clicks (Fig. S2); homozygotes produced only a few or no USIs at all, so they were not considered here. In the case of USDs, wild-types and heterozygotes of both mouse lines produced an average of 80–90% of the USDs combined with clicks, which is a significantly higher proportion than in the respective homozygous mutants of their own line (anova, *P* < 0.01 or *P* < 0.05 for the *R552H* line and *P* < 0.01 for the *S321X* line; Fig. 6a). Our earlier investigation suggested that *R552H* homozygotes produced clicks at a significantly higher rate and USDs at a significantly lower rate compared with wild-types and heterozygotes (Groszer *et al*. 2008; anova, *P* < 0.001 or *P* < 0.01; Fig. S3). We hypothesized that a click could signal the attempt to produce an ultrasound; in support of this, the average sum of USDs and clicks did not differ between *R552H* homozygotes, heterozygotes and wild-types (Groszer *et al*. 2008; Fig. S3). A corresponding pattern of results was observed for the *S321X* line, i.e. homozygotes produced significantly more clicks and less USDs than heterozygotes and wild-types (anova, *P* < 0.001) while the sums of clicks and USDs did not differ between the groups (Fig. 6b).

### Vocalization properties

#### Call duration

**Figure 7:**
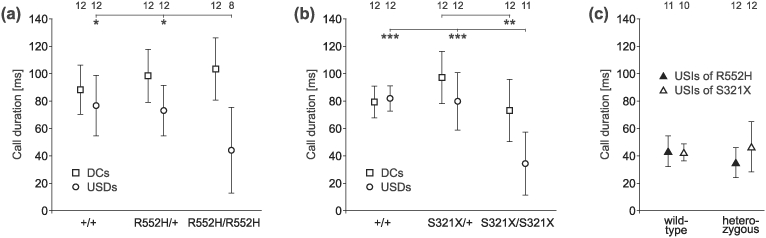
Durations of DCs and of USDs or USIs (a) The duration of DCs was similar for the three groups of the *R552H* mouse line, while *S321X* homozygotes had shorter DCs than heterozygotes (b). USDs of homozygotes of both mouse lines were shorter than those of wild-types and heterozygotes of the respective lines (a, b). There were no differences in duration between USIs of wild-type and heterozygotes of both mouse lines (c). Sounds of 8–12 animals per group could be analysed (see numbers in the top row of the figure).

Overall, the duration of DCs was similar (average 70–100 milliseconds) for the different genotypes (Fig. 7a,b). Only *S321X* heterozygotes had significantly longer DCs than the homozygotes of the same type (anova, *P* < 0.01, Fig. 7b). With only about 40 milliseconds average duration, USDs of homozygotes of both mouse lines were significantly shorter than those of wild-types and heterozygotes of the respective lines which had an average duration of almost 80 milliseconds (anova with *P* < 0.05 for the *R552H* line and *P* < 0.001 for the *S321X* line; Fig. 7a,b). The USIs of wild-type and heterozygous animals did not differ, with durations of about 40 milliseconds (Fig. 7c).

#### Durations of inter-call intervals

**Figure 8:**
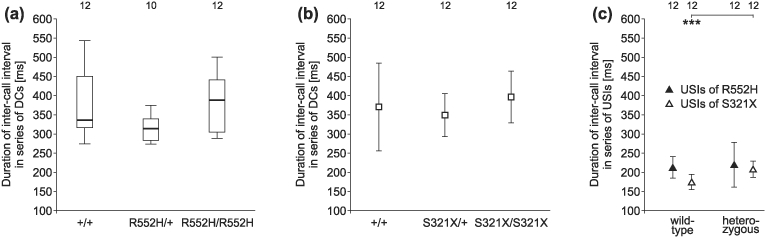
Duration of inter-call intervals in series of DCs and series of USIs (a, b) Inter-call intervals in series of DCs did not differ among the genotypes of either line. (c) Inter-call intervals in series of USIs of the *S321X* heterozygotes were longer than those of their wild-type littermates. USI inter-call intervals did not differ between *R552H* heterozygotes and their wild-type littermates. Mostly, sounds of 12 animals per group could be analysed (see numbers in the top row of the figure).

All genotypes of both mouse lines produced series of DCs with similar durations of inter-call intervals (no statistically significant differences, anova or anova on ranks) most of which were in the range of 300–400 milliseconds (Fig. 8a,b). For the *S321X* line, heterozygotes produced USI series with durations of inter-call intervals that were significantly longer than those of wild-types (*t* test, *P* < 0.001; Fig. 8c). *R552H* heterozygotes did not differ from wild-type littermates in this respect (Fig. 8c).

#### Frequency jumps

**Figure 9:**
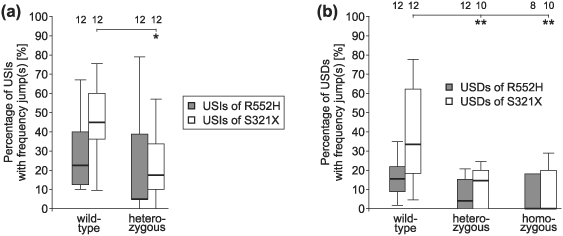
Percentages of ultrasounds with frequency jumps in isolation [USIs, (a)] or in distress [USDs, (b)] There were no differences between the genotypes of the *R552H* mouse line with regard to the percentages of frequency jumps in USIs or USDs. *S321X* wild-types, however, emitted more USIs and USDs with frequency jumps than other genotypes. Sounds of 8–12 animals per group could be analysed (see numbers in the top row of the figure).

Given that ultrasounds may end with a frequency jump (Figs 2 and 3), we compared the percentage of USIs and USDs with frequency jumps among the genotypes (Fig. 9a,b). Ultrasounds (both USIs and USDs) from wild-type animals of the *S321X* line had a significantly higher rate (about twice as high) of frequency jumps (*U* test, *P* < 0.05 or anova on ranks, *P* < 0.01) than heterozygotes and homozygotes.

#### Sound pressure level

**Figure 10:**
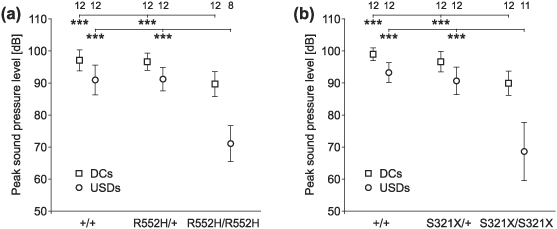
Peak SPL of DCs and USDs (a, b) DCs and USDs of homozygotes of both mouse lines were softer than the calls emitted by the respective heterozygous and wild-type animals. Sounds of 8–12 animals per group could be analysed (see numbers in the top row of the figure).

For both *R552H* and *S321X*, the DCs and USDs of homozygous mutants were significantly softer (by 10 or 20 dB, respectively) than the calls of wild-types and heterozygotes (anova, *P* < 0.001; Fig. 10a,b). USDs of wild-types and heterozygotes were significantly (by about 30 dB) louder than the respective USIs (*U* test, *P* < 0.001 or *t* test, *P* < 0.001; Fig. S4). USIs of homozygotes could not be compared because of insufficient numbers of vocalizations. Peak SPLs of DCs, USDs (Fig. 10a,b) and USIs (Fig. S4a,b) did not differ between wild-types and heterozygotes.

**Figure 11:**
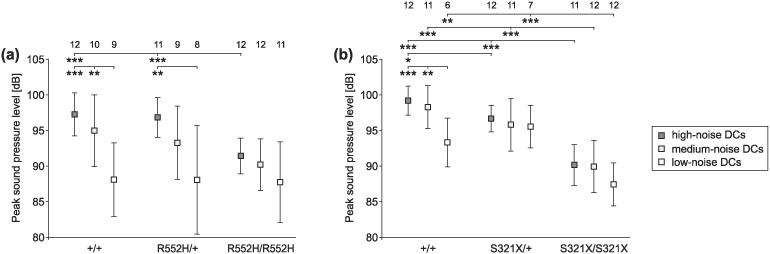
Peak SPL of DCs in relation to their noise content (a, b) In wild-types of both mouse lines and *R552H* heterozygotes, high-noise DCs were louder than the low-noise DCs. Homozygous mutants of both mouse lines produced the softest calls without significant differences in SPL between the DCs of different noise content. Sounds of 6–12 animals per group could be analysed (see numbers in the top row of the figure).

The DCs of the three different noise classes differed in their SPLs in the wild-types of both lines and heterozygous *R552H* animals but not in the other groups of animals (Fig. 11). Where significant differences occurred (anova, *P* < 0.01 or *P* < 0.001; Fig. 11), high-noise DCs were always louder than low-noise DCs, with the SPLs of the medium-noise DCs in between. Further, the DCs of all noise classes emitted by homozygous *S321X* animals were significantly softer (anova, *P* < 0.001, *P* < 0.01) than the respective DCs of the wild-types and heterozygotes (Fig. 11b). This was also the case for the high-noise DCs of the *R552H* animals (anova, *P* < 0.001; Fig. 11a).

## Discussion

Studies of *Foxp2* disruptions generated by gene targeting (Fujita *et al*. 2008; Shu *et al*. 2005) reported absence of USIs in homozygous and reduced numbers of USIs in heterozygous, 6–10 day old pups. Wild-types and heterozygotes produced clicks associated with and separated from USIs, while homozygotes produced clicks without USIs at reduced rates compared with the other genotypes (Fujita *et al*. 2008; Shu *et al*. 2005). Our data confirm that homozygous disruptions are associated with dramatic reductions in USI output. However, the heterozygotes emitted USIs at rates not significantly different from those of wild-types (Figs 4a and S1a). Notably, the prior investigations described moderate developmental delays in heterozygous animals, which were reported to have lower weight than wild-types at the age when vocalization was assessed (6–10 days) (Fujita *et al*. 2008; Shu *et al*. 2005). This key confounding factor is absent in our heterozygotes, which show no indications of developmental delay and have similar weight to wild-types (Fig. 1). The absence of such confounds may explain the distinct heterozygous vocalization findings in the different studies. Crucially, our findings do not support the previous claims (Fujita *et al*. 2008; Shu *et al*. 2005) that a complete lack of functional Foxp2 protein prevents ultrasonic vocalization. Our analyses of homozygotes in the distress situation clearly show that motor coordination for ultrasound production remains intact. The broader data indicate that connections between *Foxp2* dysfunction and changes in mouse pup vocalizations are more complex than these prior studies suggested, discussed further below.

### Homozygous mutants lacking functional Foxp2 can produce multiple call types with complex structures

Such mice emit ultrasounds, harmonically structured DCs and clicks (Figs 2–5 and S1). Thus, structural conditions and neural mechanisms underlying motor coordination of all these different vocalizations remain intact, even in total absence of functional Foxp2 protein in both *R552H* and *S321X* homozygotes.

### Total rates of distress vocalizations (DCs and USDs) are unchanged in Foxp2 mutants

Independent of genotype, mouse pups emitted similar numbers of audible DCs (Figs 4b and S1b) leading us to hypothesize that the emotional regulation for vocalizing is not altered by *Foxp2* disruption at the age of 4 days. If emotional regulation is unaltered, why should the number of emitted ultrasounds vary between genotypes (Figs 4 and S1)? Because clicks are often associated with ultrasounds (Figs 2, 3, 6 and S2), perhaps the occurrence of a click without ultrasound represents an attempted ultrasound emission, which is unsuccessful or undetectable because of insufficient energy. Consequently, the sum of emitted clicks and ultrasounds should be constant and genotype independent, as observed for USDs (Figs 6b and S3). Although we currently cannot exclude the possibility of emotional differences between genotypes, we do not believe this to be the most parsimonious explanation for our data.

### Homozygous Foxp2 mutants emit comparably soft sounds

Given that the distress data discussed above indicate unchanged attempts to vocalize, why were no USIs recorded from homozygous *R552H* mutants and only few from just 4 of the 12 *S321X* homozygotes? A potential explanation is the following. The homozygous mutants showed significantly reduced rates of postnatal weight gain accompanied by general motor dysfunction (Groszer *et al*. 2008). Evidence of subtle postnatal dilation of distal airspaces in the lungs of homozygous nulls has also been documented (Shu *et al*. 2007). Although we minimized effects of developmental delay by assessing pups at an early time-point (P4), we found significantly reduced weight (Fig. 1) and significantly lower SPLs of DCs and USDs in homozygotes compared with heterozygotes and wild-types (Fig. 10). USDs are typically much louder than USIs. The latter are, on average, just above the sensitivity limits (∼56 dB) of our recording and analysing apparatus (Fig. S4). Thus, it is plausible that homozygous mutants emitted very soft USIs undetectable with our equipment. Such an interpretation, whereby soft USIs can go undetected because of technical reasons, may also hold for other studies in which USIs of homozygous mutants appeared absent while clicks were present (Fujita *et al*. 2008; Groszer *et al*. 2008; Shu *et al*. 2005).

The lower proportion of high-noise DCs emitted by homozygotes (Fig. 5) and the lower SPLs of their high-noise DCs (Fig. 11) contributed to their comparably soft DCs (Fig. 10). Loud vocalizations tend to have high-noise content (Fitch *et al*. 2002).

**Figure 12:**
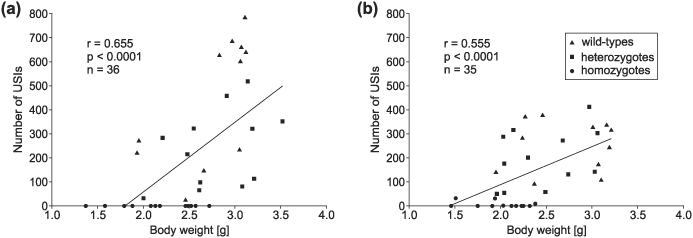
Spearman correlation analysis between body weight and number of recorded USIs of the pups The correlation coefficients (*r*) and corresponding *P* values are indicated. The regression lines (*y* = *y*_0_ + a ^*^x) with *y*_0_ = −521.9, *a* = 290.0 for the *R552H* pups (a) and *y*_0_ = −227.8, *a* = 158.2 for the *S321X* pups (b) describe these correlations. On average, the number of recorded USIs increases with increasing body weight for both mouse lines.

Taken together, low weight, small numbers of high-noise DCs and low SPLs of all vocalizations suggest that somatic weakness of homozygous mutants plays a significant role in their altered vocal output. This may yield difficulties in creating enough power for tension of the vocal cords necessary for emitting loud whistle-like ultrasounds (Roberts 1975; Sanders *et al*. 2001) and for generating the high air velocity through the throat necessary to produce loud and, therefore, noisy DCs. A statistically significant correlation between body weight and number of recorded USIs in both mouse lines (*P* < 0.0001 in each case; Fig. 12) supports the hypothesis that somatic weakness, as measured via body weight, influences the number of detectable sounds. On average, more USIs were recorded from heavier pups.

### Homozygous Foxp2 mutants emit vocalizations of normal time structure

Homozygous mutants and their wild-type littermates did not differ in the durations of DCs (Fig. 7a,b) and of the inter-call intervals in DC series (Fig. 8a,b), indicating similar overall coordination of calling in the time domain. USDs of homozygotes were shorter than USDs of heterozygous and wild-type animals (Fig. 7a,b). This finding may again relate to somatic weakness and general motor dysfunction of homozygotes, given that pups are known to emit longer ultrasounds when they are more motorically active (Branchi *et al*. 2004).

### Pups heterozygous for Foxp2 mutations do not show consistent differences in innate vocalisations

Heterozygous *R552H* mutants did not differ from wild-type littermates in any of the vocalization properties assessed. That is, the mutation in heterozygous mice had no measurable effect on innate emotional vocalizing, at least in 4-day-old pups, prior to any possible influence of auditory feedback. Because *Foxp2* is expressed in many brain areas of sensory, motor and emotional processing (Campbell *et al*. 2009), adult heterozygotes will now be investigated for possible influence of hearing and emotions on their vocalizations.

Compared with wild-type littermates, *S321X* heterozygotes showed significant differences in 5 of the 16 measures characterizing the animals' sounds: they emitted fewer USDs (Fig. 4b), fewer ultrasounds (USIs and USDs) with frequency jumps (Fig. 9), high-noise DCs with lower SPLs (Fig. 11b) and USIs with longer inter-call intervals (Fig. 8c). Thus, the *S321X* null allele mutation of *Foxp2* possibly shows differences in phenotypic effects from the *R552H* missense mutation (Vernes *et al*. 2006). Notably, however, the magnitudes of the vocalization differences in *S321X* heterozygotes were small, and even larger differences could be observed between the wild-types of the two mouse lines of our study. Genetic drift during backcrossing of the lines (Egan *et al*. 2007) as well as litter effects may account for differences between wild-types. This stresses previous findings (Brunelli 2005; Ehret 2005; Thornton *et al*. 2005) that vocalization rates and acoustic characteristics of rodent sounds are subject to numerous genetic influences.

### Concluding remarks

The present findings significantly extend previous data on vocalizations of *R552H* mutants (Groszer *et al*. 2008) by incorporating more animals, novel sound parameters and a second line of mice carrying a distinct mutation (*S321X* mutants) in the analysis, leading to the new insight that *Foxp2* expression is not an essential prerequisite for innate production of emotional vocalizations, at least in young, still deaf animals. Research in primates has provided ample evidence for the existence of separable neural circuits for the production of learned and innate vocal patterns (Jürgens 2009). In humans, neocortical damage to circuits supporting learned vocalizations completely prevents speaking or singing while innate non-verbal emotional utterances such as crying and laughing are preserved (Jürgens 2002). The present study assessed this distinction in relation to potential functions of *Foxp2* in mice. For the parameters measured, our data show that dysfunction of *Foxp2* in 4-day-old mouse pups does not overtly affect the emission of vocalization series that are innately produced in appropriate behavioural contexts, arguing against pure roles of *Foxp2* in motor production of vocalizations. Vocalization differences in homozygous *Foxp2* mutants co-occur with developmental delays, somatic weakness and general motor dysfunction, and are unlikely to represent direct effects. We also found that innate vocalizations of heterozygous pups were either unaffected (*R552H*) or showed small effects (*S321X*) that were not as large as differences between wild-types of the different lines. Any differences were in the range and of the type previously observed for normal mouse pups of different strains (Bell *et al*. 1972; D’Udine *et al*. 1982; Hahn & Schanz 2002; Hahn *et al*. 1997, 1998; Robinson & D’Udine 1982; Roubertoux *et al*. 1996). Given that other studies implicate *Foxp2* in motor-skill learning in mice (Groszer *et al*. 2008) and in auditory-guided vocal learning in songbirds (Haesler *et al*. 2007), we hypothesize that its effects on motor coordination may be most apparent in the context of learning motor patterns for vocalizing.

## Supporting Information

**Figure S1:** Number of vocalizations emitted by *R552H* mutants. (a) USIs. Wild-types and heterozygotes produced similar USI rates during the 15 min of recording, while ultrasounds from homozygous pups were not detected. (b) Number of DCs and USDs emitted in the distress situation. The rates of DCs did not depend on the genetic background. Homozygotes produced significantly less USDs than their wild-type and heterozygous littermates. All three *R552H* groups produced less USDs than DCs. Data represent an expanded sample from that reported in Groszer etal. (2008). Sounds of 11 or 12 animals per group could be analysed (see numbers in the top row of the figure).

**Figure S2:** Percentage of ultrasounds in combination with clicks emitted in isolation (USIs) by *R552H* and *S321X* pups. Except for wild-type *S321X* animals with a low percentage of USIs associated with clicks, about one third of the USIs from the other groups contained clicks. Sounds of 10 or 12 animals per group could be analysed (see numbers in the top row of the figure).

**Figure S3:** Number of ultrasounds (USDs) and clicks produced by *R552H* mutants in the distress condition. Wild-type and heterozygous pups emitted more USDs than clicks, while this was reversed in homozygotes. The sum of the emitted clicks and USDs, however, did not differ between genotypes. Data represent an expanded sample from that reported in Groszer etal. (2008). Sounds of 11 or 12 animals per group could be analysed (see numbers in the top row of the figure).

**Figure S4**: Peak SPL of USDs or USIs. USDs were louder than USIs for all genotypes. (a) Wild-type animals; (b) heterozygotes. Sounds of 11 or 12 animals per group could be analysed (see numbers in the top row of the figure).

**Table S1:** List of statistical tests with their parameters and results as applied to the data shown in the figures.

As a service to our authors and readers, this journal provides supporting information supplied by the authors. Such materials are peer-reviewed and may be re-organized for online delivery, but are not copy-edited or typeset. Technical support issues arising from supporting information (other than missing files) should be addressed to the authors.
