# Supplementary material for: The structure of innate vocalizations in Foxp2-deficient mouse pups
Source: Genes Brain Behav. 2010 Jun;9(4):390–401. doi: 10.1111/j.1601-183X.2010.00570.x (PMC2895353; doi:10.1111/j.1601-183X.2010.00570.x)
Supplement: Supplementary file 1 [file gbb0009-0390-SD1.pdf]

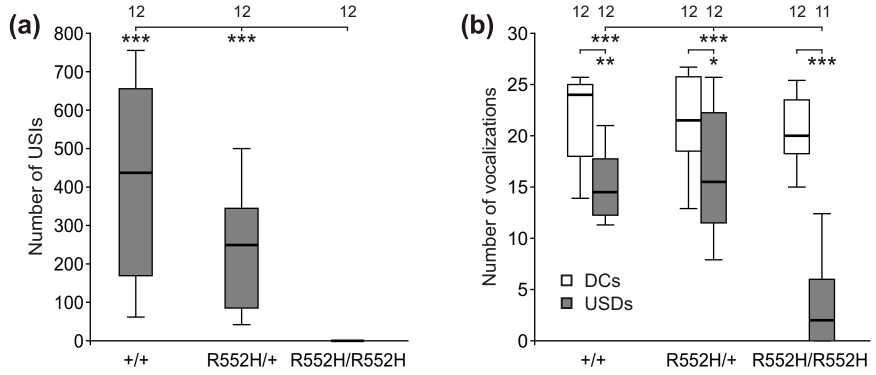

Figure S1: Number of vocalizations emitted by R552H mutants.

(a) Ultrasounds emitted in isolation (USIs). Wild-types and heterozygotes produced similar USI rates during the 15 min of recording, while homozygotes pups did not vocalize at all under these conditions.

(b) Number of distress calls (DCs) and ultrasounds in distress (USDs) emitted in the distress situation. The rates of DCs did not depend on the genetic background. Homozygotes produced significantly less USDs than their wildtype and heterozygous littermates. All three R552H groups produced less USDs than DCs. Data represent an expanded sample from that reported in Groszer et al. (2008).
