# Supplementary material for: The structure of innate vocalizations in Foxp2-deficient mouse pups
Source: Genes Brain Behav. 2010 Jun;9(4):390–401. doi: 10.1111/j.1601-183X.2010.00570.x (PMC2895353; doi:10.1111/j.1601-183X.2010.00570.x)
Supplement: Supplementary file 2 [file gbb0009-0390-SD2.pdf]

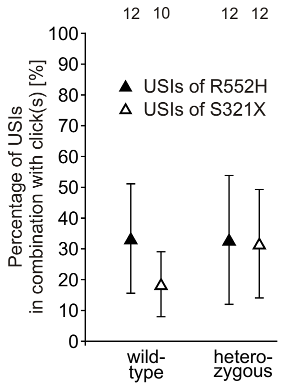

Figure S2: Percentage of ultrasounds in combination with clicks emitted in isolation (USIs) by R552H and S321X pups.

Except for wild-type S321X animals with a low percentage of USIs associated with clicks, about 1/3 of the USIs from the other groups contained clicks.

Supplement figure 2

Gaub et al.
