# Supplementary material for: The structure of innate vocalizations in Foxp2-deficient mouse pups
Source: Genes Brain Behav. 2010 Jun;9(4):390–401. doi: 10.1111/j.1601-183X.2010.00570.x (PMC2895353; doi:10.1111/j.1601-183X.2010.00570.x)
Supplement: Supplementary file 3 [file gbb0009-0390-SD3.pdf]

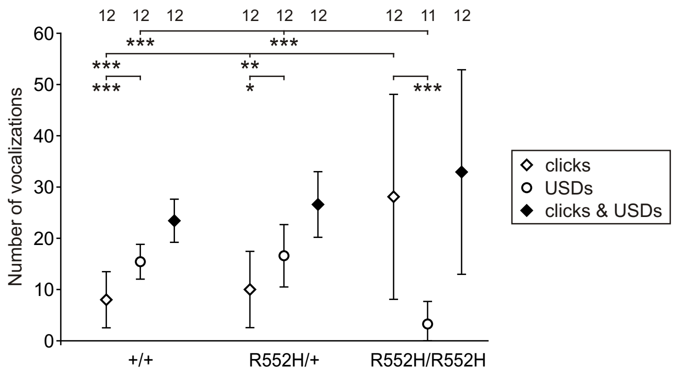

Figure S3: Number of ultrasounds (USDs) and clicks produced by R552H mutants in the distress condition.

Wild-type and heterozygous pups emitted more USDs than clicks, while this was reversed in homozygotes. The sum of the emitted clicks and USDs, however, did not differ between genotypes. Data represent an expanded sample from that reported in Groszer et al. (2008).
