# Supplementary material for: The structure of innate vocalizations in Foxp2-deficient mouse pups
Source: Genes Brain Behav. 2010 Jun;9(4):390–401. doi: 10.1111/j.1601-183X.2010.00570.x (PMC2895353; doi:10.1111/j.1601-183X.2010.00570.x)
Supplement: Supplementary file 4 [file gbb0009-0390-SD4.pdf]

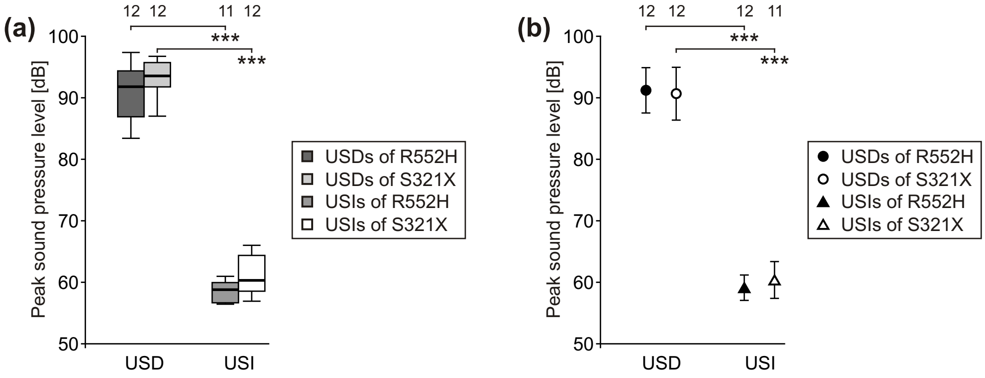

Figure S4: Peak sound pressure level of ultrasounds emitted in distress (USDs) or in isolation (USIs).

USDs were louder than USIs for all genotypes. (a) Wild-type animals, (b) heterozygotes.

Supplement figure 4

Gaub et al.
