# Supplementary material for: The structure of innate vocalizations in Foxp2-deficient mouse pups
Source: Genes Brain Behav. 2010 Jun;9(4):390–401. doi: 10.1111/j.1601-183X.2010.00570.x (PMC2895353; doi:10.1111/j.1601-183X.2010.00570.x)
Supplement: Supplementary file 5 [file gbb0009-0390-SD5.pdf]

**Table S1**

| Number of figure | mouse line | object of comparison | t-test | | | one-way ANOVA | | | | Tukey-test | one-way ANOVA on ranks | | | U-test | | $Chi^2$-test | | | Spearman Correlation | |
|---|---|---|---|---|---|---|---|---|---|---|---|---|---|---|---|---|---|---|---|---|
| | | | t-value | df | p-value | F-value | df effect | df residual | p-value | p-value | value of Kruskal-Wallis H | df | p-value | value of Mann-Whitney U | p-value | $Chi^2$-value | df | p-value | correlation coefficient | p-value |
| 1 | *R552H* | | | | | | | | | | 11.359 | 2 | 0.003 | 23.5 wt vs hom<br>21.0 het vs hom | 0.006<br>0.004 | | | | | |
| 1 | *S321X* | | | | | | | | | | 13.548 | 2 | 0.001 | 13.0 wt vs hom<br>32.5 het vs hom | < 0.001<br>0.024 | | | | | |
| 4a | *S321X* | | | | | | | | | | | | | | | 12.0<br>12.0 | 1<br>1 | < 0.001 hom vs wt<br>< 0.001 hom vs het | | |
| 4a | S321X | | | | | | | | | | 23.852 | 2 | < 0.001 | 0.0 wt vs hom<br>0.0 het vs hom | < 0.001<br>< 0.001 | | | | | |
| 4b | *S321X* | DCs | | | | 2.072 | 35 | 33 | 0.142 | | | | | | | | | | | |
| 4b | *S321X* | USDs | | | | 45.256 | 35 | 33 | < 0.001 | < 0.001 wt vs hom<br>< 0.001 het vs hom<br>0.002 wt vs het | | | | | | | | | | |
| 4b | S321X | DCs & USDs of wt<br>DCs & USDs of hom | -4.960<br>7.359 | 22<br>22 | < 0.001<br>< 0.001 | | | | | | | | | | | | | | | |
| 5a | *R552H* | DC types of wt | | | | 359.907 | 35 | 33 | < 0.001 | < 0.001 hn vs mn<br>< 0.001 hn vs ln<br>0.049 mn vs ln | | | | | | | | | | |
| 5a | *R552H* | DC types of het | | | | | | | | | 20.387 | 2 | < 0.001 | 0.0 hn vs mn<br>0.0 hn vs ln | < 0.001<br>< 0.001 | | | | | |
| 5a | *R552H* | DC types of hom | | | | 1.186 | 35 | 33 | 0.318 | | | | | | | | | | | |
| 5a | *R552H* | high-noise DCs | | | | | | | | | 22.462 | 2 | < 0.001 | 3.0 wt vs hom<br>0.0 het vs hom | < 0.001<br>< 0.001 | | | | | |
| 5a | *R552H* | medium-noise DCs | | | | | | | | | 22.706 | 2 | < 0.001 | 3.0 wt vs hom<br>0.0 het vs hom | < 0.001<br>< 0.001 | | | | | |
| 5a | *R552H* | low-noise DCs | | | | | | | | | 14.659 | 2 | < 0.001 | 15.5 wt vs hom<br>13.0 het vs hom | 0.001<br>0.001 | | | | | |
| 5b | *S321X* | DC types of wt | | | | | | | | | 27.481 | 2 | < 0.001 | 8.0 hn vs mn<br>0.0 hn vs ln<br>5.0 mn vs ln | < 0.001<br>< 0.001<br>< 0.001 | | | | | |
| 5b | *S321X* | DC types of het | | | | | | | | | 15.641 | 2 | < 0.001 | 24.5 hn vs mn<br>9.5 hn vs ln | 0.007<br>< 0.001 | | | | | |
| 5b | *S321X* | DC types of hom | | | | 0.592 | 35 | 33 | 0.559 | | | | | | | | | | | |
| 5b | *S321X* | high-noise DCs | | | | | | | | | 10.432 | 2 | 0.005 | 17.0 wt vs hom<br>31.0 het vs hom | 0.002<br>0.019 | | | | | |
| 5b | *S321X* | medium-noise DCs | | | | | | | | | 9.229 | 2 | 0.010 | 22.0 het vs hom | 0.004 | | | | | |
| 5b | *S321X* | low-noise DCs | | | | | | | | | 15.124 | 2 | < 0.001 | 8.0 wt vs hom<br>18.0 het vs hom | < 0.001<br>0.003 | | | | | |
| 6a | *R552H* | | | | | 6.900 | 31 | 29 | 0.004 | 0.003 wt vs hom<br>0.036 het vs hom | | | | | | | | | | |
| 6a | *S321X* | | | | | 7.661 | 33 | 31 | 0.002 | 0.010 wt vs hom<br>0.003 het vs hom | | | | | | | | | | |
| 6b | *S321X* | clicks | | | | 47.473 | 34 | 32 | < 0.001 | < 0.001 wt vs hom<br>< 0.001 het vs hom | | | | | | | | | | |
| 6b | *S321X* | USDs | | | | 45.256 | 35 | 33 | < 0.001 | < 0.001 wt vs hom<br>< 0.001 het vs hom<br>0.002 wt vs het | | | | | | | | | | |
| 6b | *S321X* | sum of clicks & USDs | | | | 1.994 | 35 | 33 | 0.152 | | | | | | | | | | | |
| 6b | *S321X* | clicks & USDs of wt<br>clicks & USDs of het<br>clicks & USDs of hom | -14.496<br>-5.003<br>6.436 | 22<br>21<br>22 | < 0.001<br>< 0.001<br>< 0.001 | | | | | | | | | | | | | | | |

| Number of figure | mouse line | object of comparison | t-test | | | one-way ANOVA | | | | Tukey-test | one-way ANOVA on ranks | | | U-test | | $Chi^2$-test | | | Spearman Correlation | |
|---|---|---|---|---|---|---|---|---|---|---|---|---|---|---|---|---|---|---|---|---|
| | | | t-value | df | p-value | F-value | df effect | df residual | p-value | p-value | value of Kruskal-Wallis H | df | p-value | value of Mann-Whitney U | p-value | $Chi^2$-value | df | p-value | correlation coefficient | p-value |
| 7a | *R552H* | DCs | | | | 1.759 | 35 | 33 | 0.188 | | | | | | | | | | | |
| 7a | *R552H* | USDs | | | | 5.228 | 31 | 29 | 0.012 | 0.013 wt vs hom<br>0.030 het vs hom | | | | | | | | | | |
| 7b | *S321X* | DCs | | | | 5.561 | 35 | 33 | 0.008 | 0.008 het vs hom | | | | | | | | | | |
| 7b | *S321X* | USDs | | | | 23.756 | 34 | 32 | < 0.001 | < 0.001 wt vs hom<br>< 0.001 het vs hom | | | | | | | | | | |
| 7c | *R552H* | | 1.779 | 21 | 0.090 | | | | | | | | | | | | | | | |
| 7c | *S321X* | | -0.674 | 20 | 0.508 | | | | | | | | | | | | | | | |
| 8a | *R552H* | | | | | | | | | | 5.695 | 2 | 0.058 | | | | | | | |
| 8b | *S321X* | | | | | 0.973 | 35 | 33 | 0.389 | | | | | | | | | | | |
| 8c | *R552H* | | -0.381 | 22 | 0.707 | | | | | | | | | | | | | | | |
| 8c | *S321X* | | -3.974 | 22 | < 0.001 | | | | | | | | | | | | | | | |
| 9a | *R552H* | | | | | | | | | | | | | 39.0 | 0.061 | | | | | |
| 9a | *S321X* | | | | | | | | | | | | | 28.0 | 0.012 | | | | | |
| 9b | *R552H* | | | | | | | | | | 5.853 | 2 | 0.054 | | | | | | | |
| 9b | *S321X* | | | | | | | | | | 10.714 | 2 | 0.005 | 20.0 wt vs het<br>18.5 wt vs hom | 0.009<br>0.007 | | | | | |
| 10a | *R552H* | DCs | | | | 19.005 | 35 | 33 | < 0.001 | < 0.001 wt vs hom<br>< 0.001 het vs hom<br>0.943 wt vs het | | | | | | | | | | |
| 10a | *R552H* | USDs | | | | 57.369 | 31 | 29 | < 0.001 | < 0.001 wt vs hom<br>< 0.001 het vs hom<br>0.989 wt vs het | | | | | | | | | | |
| 10b | *S321X* | DCs | | | | 27.674 | 35 | 33 | < 0.001 | < 0.001 wt vs hom<br>< 0.001 het vs hom<br>0.174 wt vs het | | | | | | | | | | |
| 10b | *S321X* | USDs | | | | 58.171 | 34 | 32 | < 0.001 | < 0.001 wt vs hom<br>< 0.001 het vs hom<br>0.545 wt vs het | | | | | | | | | | |
| 11a | *R552H* | DC types of wt | | | | 11.570 | 30 | 28 | < 0.001 | < 0.001 hn vs ln<br>0.006 mn vs ln | | | | | | | | | | |
| 11a | *R552H* | DC types of het | | | | 6.396 | 27 | 25 | 0.006 | 0.004 hn vs ln | | | | | | | | | | |
| 11a | *R552H* | high-noise DCs | | | | 16.350 | 34 | 32 | < 0.001 | < 0.001 wt vs hom<br>< 0.001 het vs hom | | | | | | | | | | |
| 11b | *S321X* | DC types of wt | | | | 9.689 | 28 | 26 | < 0.001 | < 0.001 hn vs ln<br>0.004 mn vs ln | | | | | | | | | | |
| 11b | *S321X* | high-noise DCs | | | | 48.235 | 34 | 32 | < 0.001 | < 0.001 wt vs hom<br>< 0.001 het vs hom<br>0.026 wt vs het | | | | | | | | | | |
| 11b | *S321X* | medium-noise DCs | | | | 17.536 | 33 | 31 | < 0.001 | < 0.001 wt vs hom<br>0.001 het vs hom | | | | | | | | | | |
| 11b | *S321X* | low-noise DCs | | | | 17.044 | 24 | 22 | < 0.001 | 0.003 wt vs hom<br>< 0.001 het vs hom | | | | | | | | | | |
| 12a | *R552H* | | | | | | | | | | | | | | | | | | 0.655 | < 0.0001 |
| 12b | *S321X* | | | | | | | | | | | | | | | | | | 0.555 | < 0.0001 |

| Number of figure | mouse line | object of comparison | t-test | | | one-way ANOVA | | | | Tukey-test | one-way ANOVA on ranks | | | U-test | | $Chi^2$-test | | | Spearman Correlation | |
|---|---|---|---|---|---|---|---|---|---|---|---|---|---|---|---|---|---|---|---|---|
| | | | t-value | df | p-value | F-value | df effect | df residual | p-value | p-value | value of Kruskal-Wallis H | df | p-value | value of Mann-Whitney U | p-value | $Chi^2$-value | df | p-value | correlation coefficient | p-value |
| S1a | *R552H* | | | | | | | | | | 25.466 | 2 | < 0.001 | 0.0 wt vs hom<br>0.0 het vs hom | < 0.001<br>< 0.001 | | | | | |
| S1a | *R552H* | | | | | | | | | | | | | | | 24.0<br>24.0 | 1<br>1 | < 0.001 hom vs wt<br>< 0.001 hom vs het | | |
| S1a & 1 | both | USIs of hom | | | | | | | | | | | | | | 4.8 | 1 | 0.030 | | |
| S1b | *R552H* | DCs | | | | | | | | | 1.975 | 2 | 0.372 | | | | | | | |
| S1b | *R552H* | USDs | | | | | | | | | 19.129 | 2 | < 0.001 | 5.0 wt vs hom<br>4.5 het vs hom | < 0.001<br>< 0.001 | | | | | |
| S1b | *R552H* | DCs & USDs of wt<br>DCs & USDs of het<br>DCs & USDs of hom | | | | | | | | | | | | 18.5<br>37.5<br>0.0 | 0.002<br>0.050<br>< 0.001 | | | | | |
| S2 | *R552H* | | 0.053 | 22 | 0.959 | | | | | | | | | | | | | | | |
| S2 | *S321X* | | -2.069 | 20 | 0.052 | | | | | | | | | | | | | | | |
| S3 | *R552H* | clicks | | | | 9.085 | 35 | 33 | < 0.001 | 0.001 wt vs hom<br>0.004 het vs hom | | | | | | | | | | |
| S3 | *R552H* | USDs | | | | 27.032 | 34 | 32 | < 0.001 | < 0.001 wt vs hom<br>< 0.001 het vs hom | | | | | | | | | | |
| S3 | *R552H* | sum of clicks & USDs | | | | 1.844 | 35 | 33 | 0.174 | | | | | | | | | | | |
| S3<br>S3<br>S3 | *R552H*<br>*R552H*<br>*R552H* | clicks & USDs of wt<br>clicks & USDs of het<br>clicks & USDs of hom | -3.986<br>-2.374<br>4.020 | 22<br>22<br>21 | < 0.001<br>0.027<br>< 0.001 | | | | | | | | | | | | | | | |
| S4a | *R552H* | USDs & USIs of wt | | | | | | | | | | | | 0.0 | < 0.001 | | | | | |
| S4a | *S321X* | USDs & USIs of wt | | | | | | | | | | | | 0.0 | < 0.001 | | | | | |
| S4b | *R552H* | USDs & USIs of het | 26.337 | 22 | < 0.001 | | | | | | | | | | | | | | | |
| S4b | *S321X* | USDs & USIs of het | 19.447 | 21 | < 0.001 | | | | | | | | | | | | | | | |

Abbreviations: df: degrees of freedom wt: wild-types het: heterozygotes hom: homozygotes vs: versus hn: high-noise DCs mn: medium-noise DCs ln: low-noise DCs
